# Bindin is essential for fertilization in the sea urchin

**DOI:** 10.1073/pnas.2109636118

**Published:** 2021-08-16

**Authors:** Gary M. Wessel, Yuuko Wada, Mamiko Yajima, Masato Kiyomoto

**Affiliations:** ^a^Department of Molecular and Cellular Biology & Biochemistry, Division of BioMed, Brown University, Providence, RI 02912;; ^b^Tateyama Marine Laboratory, Marine and Coastal Research Center, Ochanomizu University, Tateyama 294-0301, Japan

**Keywords:** fertilization, bindin, sea urchin, CRISPR/Cas9

## Abstract

Species-specific sperm−egg interactions are essential for sexual reproduction. Broadcast spawning of marine organisms is under particularly stringent conditions, since eggs released into the water column can be exposed to multiple different sperm. Bindin isolated from the sperm acrosome results in insoluble particles that cause homospecific eggs to aggregate, whereas no aggregation occurs with heterospecific eggs. Therefore, Bindin is concluded to play a critical role in fertilization, yet its function has never been tested. Here we report that Cas9-mediated inactivation of the *bindin* gene in a sea urchin results in perfectly normal-looking embryos, larvae, adults, and gametes in both males and females. What differed between the genotypes was that the *bindin*^−/−^ sperm never fertilized an egg, functionally validating Bindin as an essential gamete interaction protein at the level of sperm–egg cell surface binding.

Sperm and eggs are two of the few cell types in an organism that fuse with each other and do so in a species-specific mechanism (1). The molecular basis for the selectivity in binding and fusion of eggs and sperm is key for fitness of an organism and is a centerpiece of speciation (2). Bindin was discovered in the sperm acrosome of the sea urchin, and intense study of this protein has resulted in many hypotheses as to its role in fertilization, and in speciation resulting from positively selected changes in its sequence (3–6). Despite the number of biochemical studies conducted, Bindin has never been directly tested for its function in fertilization. Recent progress in targeted genome manipulation through CRISPR-Cas9 and advances in sea urchin husbandry now make this goal possible.

## Results

Hpbase (https://cell-innovation.nig.ac.jp/Hpul/) was used to identify the sequence and genomic locus of the *bindin* gene from *Hemicentrotus pulcherrimus* (Hp). Four gRNAs were designed to target the pro-protein region of the predicted *bindin* gene (*SI Appendix, Materials and Methods*) with the logic that, were a mutation to disrupt the open reading frame of the cognate transcript, no parts of the mature Bindin protein would be made to complicate any interpretation of results. Each of the gRNAs along with the messenger RNA (mRNA) encoding Cas9 were injected into freshly fertilized eggs of Hp. Ensuing development was indistinguishable from uninjected, or irrelevant gRNA injections, and larvae and adults formed as normal. Our analysis focused on the sperm from eight males that were 25 mm in diameter or larger and sexually mature. Tube feet were collected from each adult, and the DNA was isolated and subjected to PCR sequencing for genotyping. Of the eight males tested, one (Bindin #2) had no mutations at any of the gRNA sites. The remaining animals had mutations in at least one of the gRNA sites, three of them in two of the gRNA sites (Bindins #1, #3, and #5), but, of those seven, mutations in two of the seven animals (Bindins #6 and #8) did not disrupt the open reading frame of the Bindin protein. Of the three adult females evaluated, each of them had a frame shift mutation in one of the gRNA sites, and the third adult (Bindin female #3) had two mutations. Overall, of the 11 adults tested, only one did not have a *bindin* mutation (Bindin #2), for a 91% efficiency of mutation, and, since no change occurred in the open reading frame of two animals, the knockout (KO) rate was 73%. The amount and morphology of *bindin*^−/−^ eggs was normal, and they fertilized and developed normally. Sperm collected from each *bindin*^−/−^ adult also appeared normal and were tested for Bindin protein expression.

**Fig. 1.**
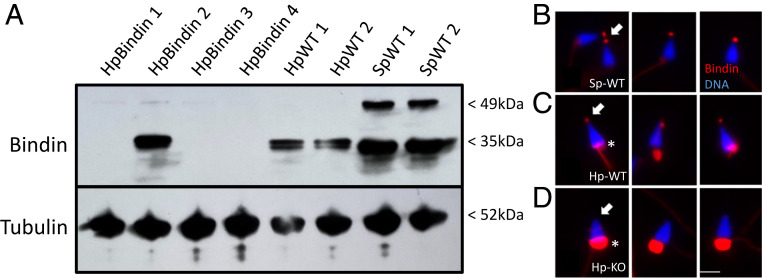
(*A*) Anti-bindin antibody reveals gene KOs in sperm. Sperm proteins were isolated, separated by sodium dodecyl sulfate polyacrylamide gel electrophoresis, blotted to nitrocellulose, and challenged by antibody incubation. Anti-tubulin antibodies were used to estimate relative loading levels of sperm protein. HpBindin represent sperm isolated from different individuals of *bindin* gRNA/Cas9 mRNA microinjections, whereas Hp-WT and Sp-WT are from WT sperm of Hp (Hp-WT) and *S. purpuratus* (Sp-WT). (*B–D*) Immunolabeling of sperm in situ with anti-bindin antibodies. (*B*) *S. purpuratus* sperm. Note the Bindin spot at the tip of the sperm (arrow). (*C*) Hp-WT sperm. Note Bindin at the tip of the sperm (arrow), and also background labeling of the midpiece (asterisk). Surprisingly, any intact IgG (containing an Fc region) binds to the midpiece in Hp, but the Fab used as secondary antibodies does not. (*D*) Hp *bindin* KO sperm. Note that there is no Bindin label at the tip of the sperm (arrow), even though background labeling of the midpiece is present (asterisk). (Scale bar, 4 μm.)

Sperm proteins from Bindin KO and wild-type (WT) animals were analyzed by immunoblotting with three different antibodies generated to the mature Bindin domain. Each of the affinity-purified antibodies had the same immunoreactivity, although AbPAV had the highest titer and was used for further studies. Immunoblots of WT sperm showed a band at ∼35 kDa in Hp and in *Strongylocentrotus purpuratus* (Fig. 1*A*). *S. purpuratus* also showed a larger band of ∼49 kDa that may reflect the pro-form of the protein. Protein from four different animals microinjected with *bindin* gRNAs/Cas9 was compared to WT animals. No Bindin protein was detectable in Bindin animals #1, #3, and #4, whereas normal amounts and size of Bindin were seen in Bindin animal #2, the only animal of these four that had no mutation.

The anti-bindin antibody was also tested in situ, first on *S. purpuratus* sperm, the species for which Bindin was originally identified (Fig. 1*B*). The antibody very selectively labeled the acrosomal vesicle at the tip of the sperm head. The antibody labeled the same structure in WT Hp also, but also resulted in signal in the midpiece (Fig. 1*C*). This midpiece signal appeared to be due to the Fc region of the intact primary antibody, since each intact antibody we used (including tubulin and irrelevant antibodies) bound to the same structure but not the Fab secondary antibodies. However, the acrosomal tip of the *bindin* KO sperm was devoid of label (Fig. 1*D*).

**Fig. 2.**
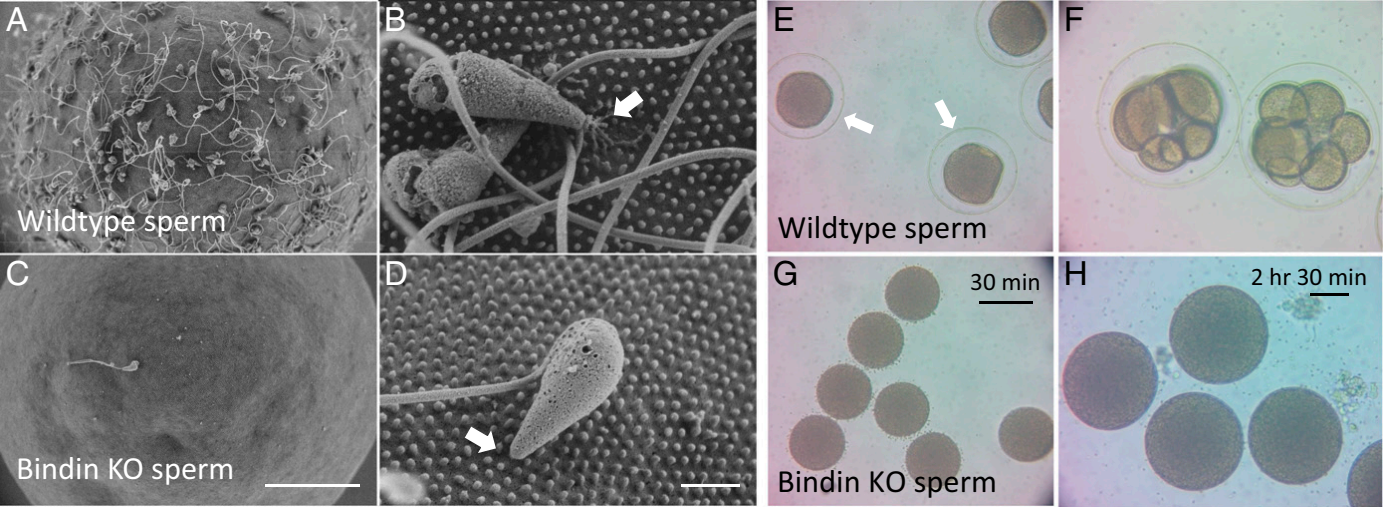
Scanning electron micrographs of (*A* and *B*) WT Hp sperm and eggs and (*C* and *D*) *bindin*^−/−^ sperm on WT eggs. Note the binding to microvilli in *B* and lack thereof in *D *(arrow). (Scale bar in *A* and *C*, 20 μm; scale bar in *B* and *D*, 2 μm.) (*E–H*) Hp *bindin*^−/−^ sperm do not bind to nor activate eggs. WT eggs challenged with WT sperm (*E*) or with *bindin*^−/−^ sperm (*G*) after 30 min of incubation show a robust fertilization envelope (arrow in *E*) with WT sperm but not with *bindin*^−/−^ sperm. After 2.5 h of incubation, the *bindin*^−/−^ sperm still do not activate eggs (*H*), whereas sibling eggs challenged with WT sperm have developed to the eight-cell stage (*F*). Scale bar in *G* = 100 micrometers; scale bar in *H* = 50 micrometers.

We examined sperm by scanning electron microscopy (SEM) and found that the *bindin* KO sperm underwent the acrosome reaction but did not bind to the egg, and their egg interaction seldom withstood the fixation and preparatory steps for SEM analysis (Fig. 2 *A*–*D*).

Bindin KO sperm never fertilized an egg (Fig. 2 *G* and *H* and Movies S1–S4). The *bindin* KO sperm were seen to reach an egg, and even bounded on and off the egg, but no sperm−egg fusion was detected based on formation of the fertilization envelope, even if left for hours when the WT fertilized embryos had accomplished several cell divisions (compare Fig. 2 *F* and *H*).

## Discussion

As essential as it is for sexual reproduction, the mechanisms of fertilization remain obscure. While many candidates have been offered as gene products essential for direct sperm−egg membrane interactions, few candidates in invertebrates have withstood the test of genetic KOs. One of particular interest is Spe-45 from the roundworm *Caenorhabditis elegans*. Spe-45 is essential in *C. elegans* sperm for fertilization, and it has structural similarity to the mammalian sperm factor, Izumo, which binds to Juno on the mammalian egg cell surface (7, 8). Each of these proteins has a transmembrane domain, and an Ig-like extracellular domain essential for fertilization, a theme recently expanded with the finding of additional mammalian sperm proteins with Ig domains essential for fertilization, for example, Spaca6 (9). Bindin is distinct from these candidates, however, in that it does not have a transmembrane domain or an Ig-like domain. Instead, retention of Bindin on the sperm cell surface following the acrosome reaction appears to rely on its insolubility (3), and perhaps its phospholipid binding (10), enabling it to link sperm and egg membranes for a subsequent fusion step of unknown mechanism.

What is the cognate egg surface molecule recognized by sperm Bindin? The search for an egg receptor for Bindin on sea urchin eggs resulted in the identification of two high molecular weight molecules, Ebr1 (Egg Bindin receptor 1) and a 350-kDa glycoprotein protein (11, 12). Ebr1 has multiple repeats of CUB domains (Complement C1s/C1r, Uegf, BMP1) contiguous with a TSP domain (Thrombospondin-1) as well as multiple hyalin (HYR) repeats, all of which are involved in extracellular and membrane-associated functions. Thus, even though Ebr1 does not have any predicted transmembrane domains that could directly instigate a fusion mechanism for sperm, Ebr1 is replete with protein interaction domains, and does appear to have species specificity, as might be expected for binding sperm.

Embryos lacking Bindin activity are capable of normal embryogenesis, larval development, metamorphosis, and sperm production indistinguishable from WT adults. While mRNA expression of *bindin* is greatest in the testis, embryos and early larvae have detectable Bindin transcript levels. These mRNA, however, appear dispensable for a normal life cycle in Hp.

The results here do lay the groundwork for more focused tests of Bindin function. High-efficiency Cas9/gRNA targeting and DNA cleavage seen here encourages a test of the B18 theoretical fusion site in Bindin (13) and replacement of Bindin segments with those of a heterospecific sperm sequence to determine the sites that control species specificity.

## Materials and Methods

The gRNAs were designed and synthesized according to CRISPRscan (www.crisprscan.org), targeting the proprotein domain of the Hp *bindin* gene at HpBase (https://cell-innovation.nig.ac.jp/Hpul/ (14)). The gRNAs were mixed with 500 ng/μL of Cas9 mRNA and injected into freshly fertilized eggs (15). The resultant embryos, larvae, and juveniles were cultured at 15 °C and achieved sexual maturation in ∼1.5 y (15). Bindin-null animals were identified both by genomic DNA sequence and by antibodies made to bindin.

## Supplementary Material

Supplementary File

Supplementary File

Supplementary File

Supplementary File

Supplementary File

## Data Availability

All study data are included in the article, *SI Appendix*, and Movies S1–S4.
