# Supplementary material for: Bindin is essential for fertilization in the sea urchin
Source: Proc Natl Acad Sci U S A. 2021 Aug 16;118(34):e2109636118. doi: 10.1073/pnas.2109636118 (PMC8403831; doi:10.1073/pnas.2109636118)
Supplement: Supplementary File [file pnas.2109636118.sapp.pdf]

Supporting information – Video legend

Supplemental Video: Hp-*bindin* -/- sperm do not bind to nor activate eggs. A and C, Wildtype eggs challenged with wildtype sperm after 30 incubation shows robust fertilization envelope with wildtype sperm but not with *bindin* -/- sperm. After 2.5 hrs of incubation, the *bindin* -/- sperm still do not activate eggs (D), whereas sibling eggs challenged with wildtype sperm have developed to the 8-cell stage already (B).

Materials and Methods

Animal culture: Eggs and sperm were collected from spawning wild-type adults of *Hemicentrotus pulcherrimus* by injection of 2mM acetylcholine into the coelomic cavity of the adult. The resultant embryos were cultured at 15°C and the larvae were fed the diatom *Chaetoceros gracilis, ad libitum*. Metamorphosis was induced by adding pieces of plastic plate covered with calcareous red algae (1). The resulting juveniles were cultured at 15°C and fed dried seaweed *Undaria pinnatifida ad libitum*. Animals achieved sexual maturation in approximately 1.5 years.

Cas9 mRNA /Guide RNAs (gRNAs) preparation and microinjection: Guide RNAs (gRNAs) were designed using CRISPRscan (www.crisprscan.org) to coding sequences of the pro-protein domain of the Hp *bindin* gene at HpBase (http://cell-innovation.nig.ac.jp/Hpul/; (2)) and synthesized as reported (3). gRNA positions and sequences were as follows:

Hp bindin 82-105 (5′-GGGGCAGTCAGTAGGGGAGT-3′)

Hp bindin 325-348 (5′-GGTTCAGTAACTGCCAGCAG-3′)

Hp bindin 373-396 (5′-GGAGCGATTAATGAAGGGTC-3′)

Hp bindin 455-478 (5′- GGGCGATGATGTCAGCAAAA-3′).

The plasmid pCS2-3xFLAG-NLS-SpCas9-NLS was a gift from Yonglong Chen (Addgene plasmid #51307), and was linearized with NotI and transcribed with SP6. This transcript encodes Cas9 (codon optimized for mammalian cells) along with two nuclear localization sequences (NLS; (4)) and has been shown previously to be functional in sea urchin embryos (5). The gRNAs were synthesized by T7 RNA polymerase using the MegaShortScript T7 transcription kit (AM1354, ThermoFisher, Waltham, MA) as described in CRISPRscan (www.crisprscan.org). The gRNAs were then purified using the

miRNeasy Mini kit (217004, Qiagen, Valencia, CA). The four gRNAs (200 ng/ul of each gRNA) were mixed with 500ng/μl of Cas9 mRNA, injected into freshly fertilized eggs as described previously in (6).

Identification of Genomic Mutations: Genomic DNA was isolated from several tube feet donated by each subject using 100 microliters of QuickExtract DNA Extraction Solution (http://www.epibio.com/) according to manufacturer's instructions. One microliter of the extraction mix was then subjected to PCR amplification of the targeted genomic DNA region: 95°C, 3 minutes, 95°C, 15 seconds, 60°C, 15 seconds, 72°C, 30 seconds, 95°C, 15 seconds, repeated 30 rounds. Primers to the genomic regions flanking the gRNA target site were used to amplify the locus. Sequence of the PCR population was accomplished using the same amplification primers and mutation sites were identified by either direct sequence or by decomposition of trace chromatograms (https://tide.deskgen.com/; (7)) or by individual clones of the gDNA amplicons.

Antibody generation and immunolabeling: Antibodies were made to three independent sites (KAV [KAVLGATKIDLPVD], HLR [HLRHHSNLLANIGD], PAV [PAVREQVLSAMQEE]; Genscript, Piscataway, NJ) of the mature Bindin sequence using two rabbits per peptide. Antibodies were isolated from the resulting antisera and affinity purified to the original immunogens. Each of the affinity purified antibodies gave the same results, although the titer of PAV was higher than the others, and the results of using the PAV-antibodies are shown here. Immunoblots were performed on *H.pulcherrimus* wildtype and Bindin mutant sperm as well as from sperm of *S. purpuratus*. Sperm protein was isolated following instruction from Professor Victor Vacquier. With such large amounts of DNA in a sperm population, simple lysis of the sperm results in highly viscous gels that run aberrantly on SDS-PAGE. So, we followed the advice of Professor Vacquier. Briefly, add 5% SDS to a sperm pellet and mix well. Add 2.5 vol of 100% ethanol and gently mix, which causes formation of a condensed gel of DNA that can be removed with forceps. Spin out the remaining supernatant at highspeed in a microfuge (10 minutes), and the pellet is largely the SDS soluble sperm protein. Wash the pellet in 75% ethanol and dissolve in a small amount of 5% SDS. Measure the protein amount and precipitate the soluble protein again by adding 2.5 vol 100% ethanol followed by precipitation. Dissolve the pellet in SDS-sample buffer with mercaptoethanol and bromophenol blue. Heat to boiling for 2 minutes and run 2 micrograms for Bindin immunoblots. The separated proteins in the gels then were transferred to nitrocellulose and immunoblotting was conducted as described (8). Anti-PAV was used at 1/5,000 dilution (0.18 micrograms/milliliter and goat anti-rabbit-HRP

(ThermoFisher Cat# PI31466) in 50% glycerol at 1/5,000 dilution (0.10 microgram/milliliter). Monoclonal anti-tubulin (12G10; Developmental Studies Hybridoma Bank; dshb.biology.uiowa.edu) was used at 1/5,000 dilution, and rabbit anti-mouse HRP (ThermoFisher Cat# PI31450) stored at -20°C in 50% glycerol was used at 1/2,000 dilution (0.20 microgram/milliliter). Signals were detected by use of the Pierce ECL Western Blotting Substrate (Cat# 32106) and captured by use of X-ray film.

Functional testing sperm in fertilization: Wildtype and *bindin* -/- sperm were diluted in filtered sea water from 100 sperm to 10 million sperm per milliliter. The same number of eggs were added to each sperm dilution and following 30 minutes incubation, the percent of fertilized eggs under each condition was measured based on presence of a fertilization envelope. The functional test was performed blind of the genotype of the animals resulting from Cas9/bindin gRNA injections. Video recordings were taken of representative samples (SI Video).

1. M. Yajima, M. Kiyomoto, Study of larval and adult skeletogenic cells in developing sea urchin larvae. *The Biological bulletin* **211**, 183-192 (2006).
2. S. Kinjo, M. Kiyomoto, T. Yamamoto, K. Ikeo, S. Yaguchi, HpBase: A genome database of a sea urchin, Hemicentrotus pulcherrimus. *Development, growth & differentiation* **60**, 174-182 (2018).
3. M. A. Moreno-Mateos *et al.*, CRISPRscan: designing highly efficient sgRNAs for CRISPR-Cas9 targeting in vivo. *Nature methods* **12**, 982-988 (2015).
4. X. Guo *et al.*, Efficient RNA/Cas9-mediated genome editing in Xenopus tropicalis. *Development* **141**, 707-714 (2014).
5. S. Shevidi, A. Uchida, N. Schudrowitz, G. M. Wessel, M. Yajima, Single nucleotide editing without DNA cleavage using CRISPR/Cas9-deaminase in the sea urchin embryo. *Developmental dynamics : an official publication of the American Association of Anatomists* **246**, 1036-1046 (2017).
6. G. M. Wessel, M. Kiyomoto, T. L. Shen, M. Yajima, Genetic manipulation of the pigment pathway in a sea urchin reveals distinct lineage commitment prior to metamorphosis in the bilateral to radial body plan transition. *Sci Rep* **10**, 1973 (2020).
7. E. K. Brinkman, T. Chen, M. Amendola, B. van Steensel, Easy quantitative assessment of genome editing by sequence trace decomposition. *Nucleic acids research* **42**, e168-e168 (2014).
8. E. Voronina, W. F. Marzluff, G. M. Wessel, Cyclin B synthesis is required for sea urchin oocyte maturation. *Developmental biology* **256**, 258-275 (2003).
